# Supplementary material for: The Cavin-1/Caveolin-1 interaction attenuates BMP/Smad signaling in pulmonary hypertension by interfering with BMPR2/Caveolin-1 binding
Source: Commun Biol. 2024 Jan 5;7:40. doi: 10.1038/s42003-023-05693-2 (PMC10770141; doi:10.1038/s42003-023-05693-2)
Supplement: Supplementary file 2 — Supplementary Figure [file 42003_2023_5693_MOESM2_ESM.pdf]

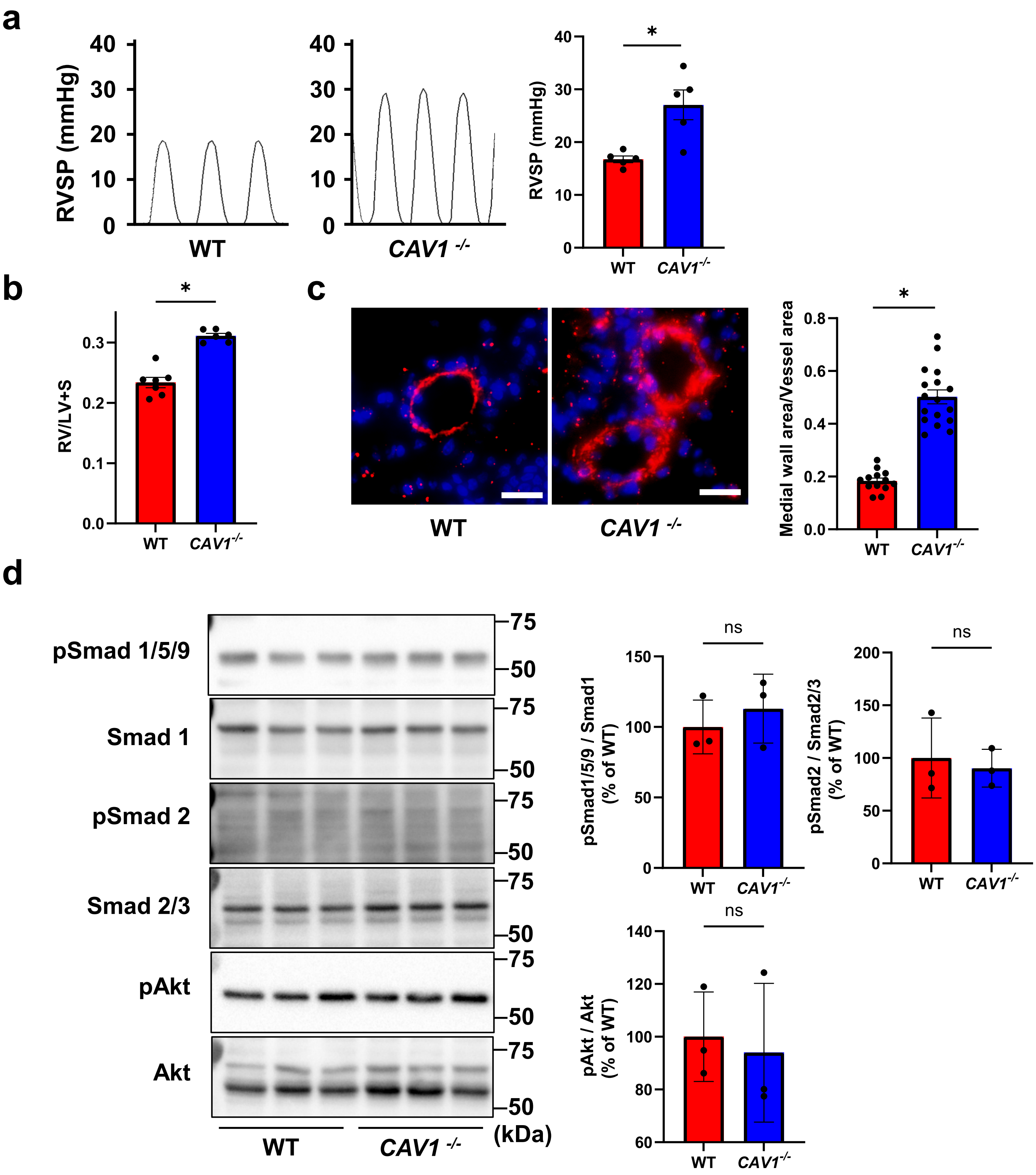

Supplementary Figure 1. ***CAV1***$^{-/-}$ **mice reveals pulmonary hypertension.**
(a) RV systolic pressure (RVSP) in WT mice and *CAV1*$^{-/-}$ mice at the age of 16 weeks under normoxic conditions. Open-chest RV catheterization using a 1.2-F pressure catheter was performed during anesthesia with 1.5% isoflurane. *P<0.05 between compared with WT mice. The data are shown as means ± sem. (b) Relative RV weight was determined as the ratio of the RV weight to LV and septum weights (RV/LV+S). *P<0.05 compared with WT mice. The data are shown as means ± sem. (c) Pulmonary vascular remodeling was assessed by measuring the medial thickness of distal pulmonary vessels from lung sections immunostained with anti-αSMA antibody. Percent wall thickness is expressed as the medial wall area divided by the area of the vessel. *P<0.05 compared with WT mice. The data are shown as means ± sem. Scale bar, 20 μm. (d) BMP/TGF-β signaling pathway and Akt phosphorylation were assessed in the lung of WT and *CAV1*$^{-/-}$ mice. The data are shown as means ± sem. ns, not significant.

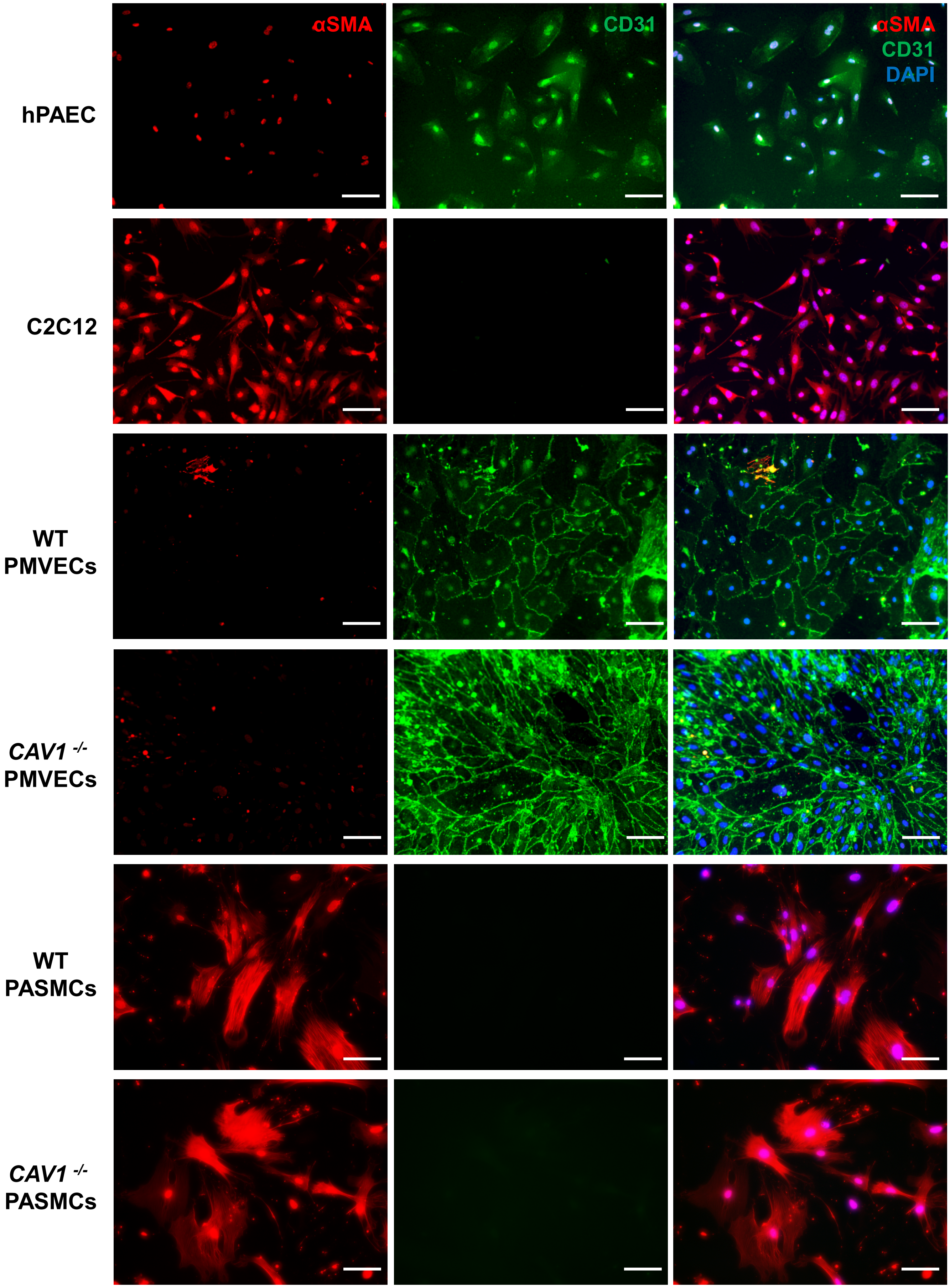

Supplementary Figure 2. Representative immunostaining images of PMVECs and PASMCs. Human pulmonary artery endothelial cells (hPAECs), C2C12 immortalized mouse myoblast cell line, PMVECs isolated from the lung of WT and *CAV1*$^{-/-}$ mice, and PASMCs isolated from the lung of WT and *CAV1*$^{-/-}$ mice were immunostained with anti-αSMA and anti-CD31 antibodies. Scale Bar, 100 μm.

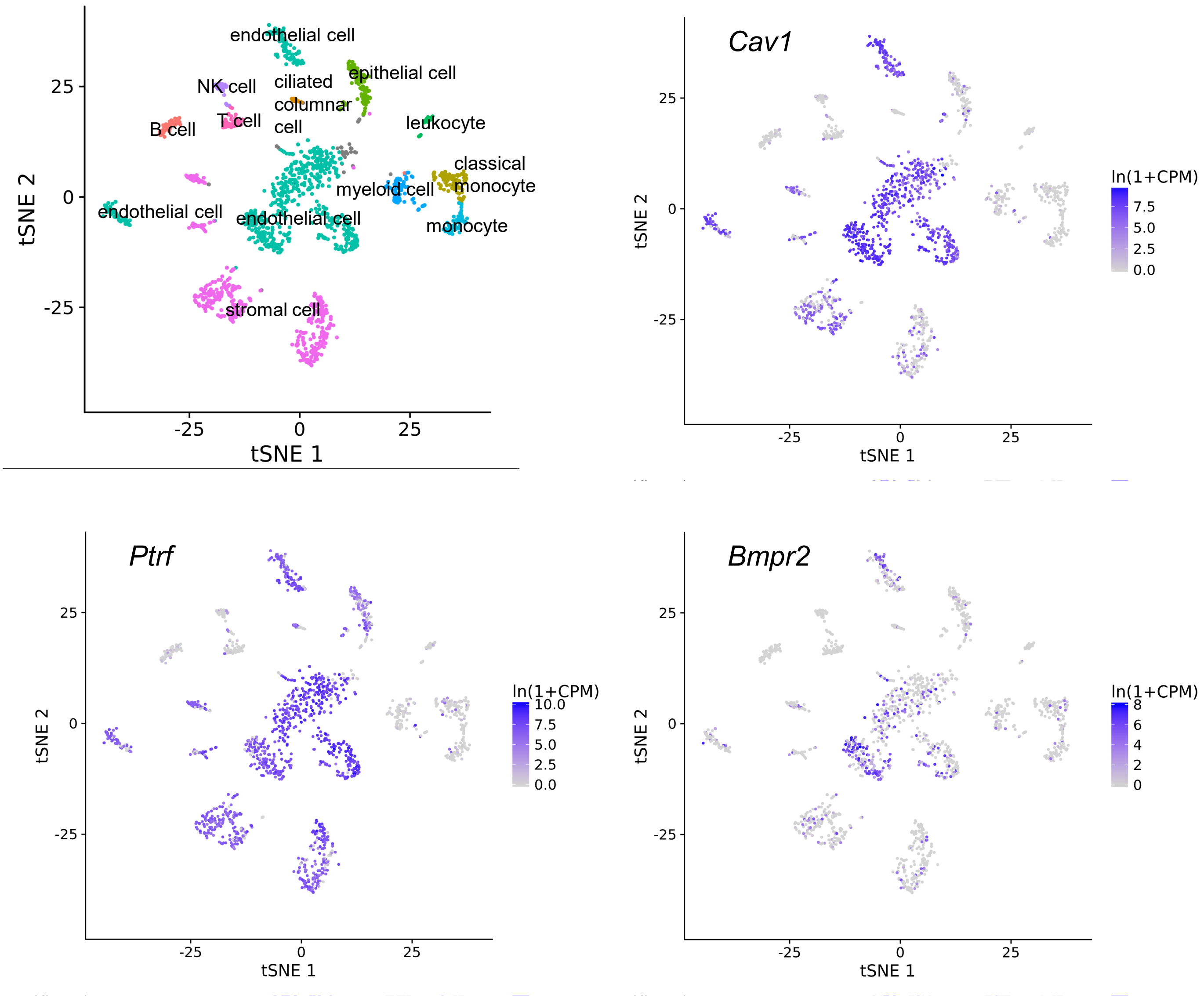

Supplementary Figure 3. Single-cell RNA sequencing from mouse lung reveals a large proportion of Bmpr2 expression in endothelial cell.
These data were analyzed from Tabula Muris portal (https://tabula-muris.ds.czbiohub.org/). Cav1, Cavin-1, and Bmpr2 expression in mouse lung were investigated by single cell RNA sequencing.

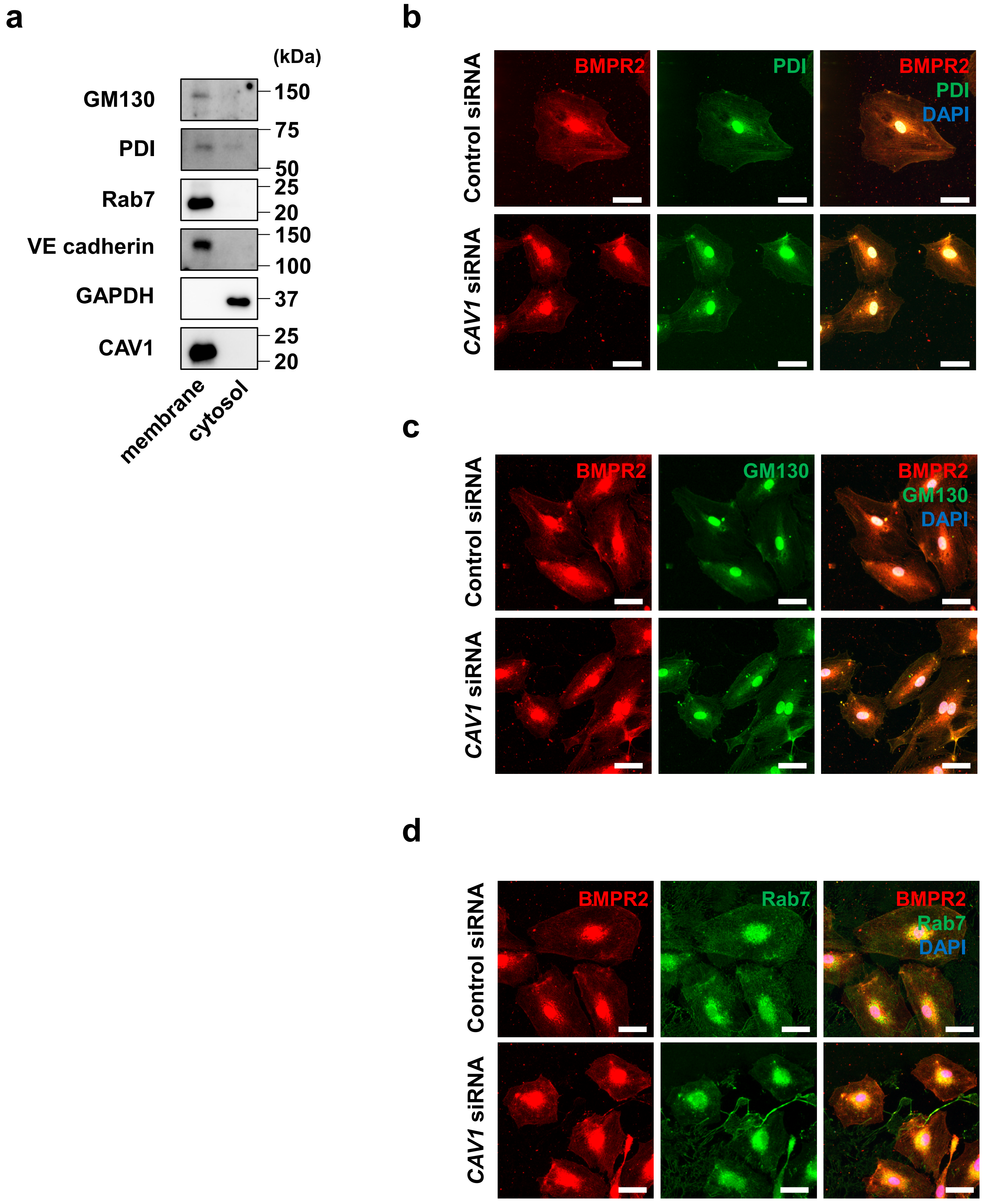

Supplementary Figure 4. Membrane and cytosol fractions separated by sucrose gradient, and immunostaining images of BMPR2 and organelles in PAECs.
(a) Membrane and cytosol fractions were separated by sucrose gradient, and lysates of these fractions were immunoblotted. GAPDH was used as a cytosolic marker and VE-cadherin was used as a membrane marker. (b) Representative fluorescent images of BMPR2 and PDI as an ER marker, GM130 as a Golgi marker (c), and Rab7 as an endosome marker (d) in control and CAV1-knocked down hPAECs. Scale bar, 50 μm.

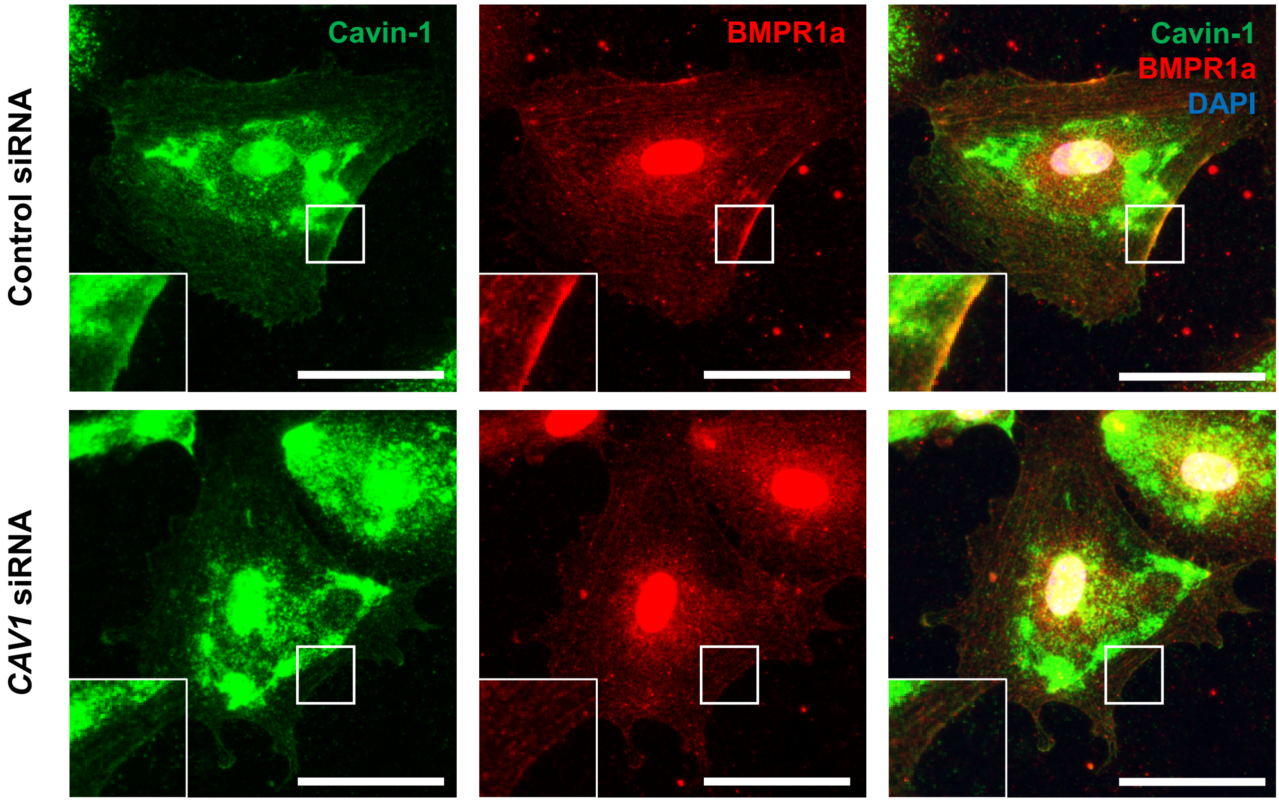

Supplementary Figure 5. Representative immunostaining images of Cavin-1 and BMPR1a in CAV1-knocked down PAECs.

Representative fluorescent images of Cavin-1 and BMPR1a in control and CAV1-knocked down hPAECs. Scale bar, 50 µm.

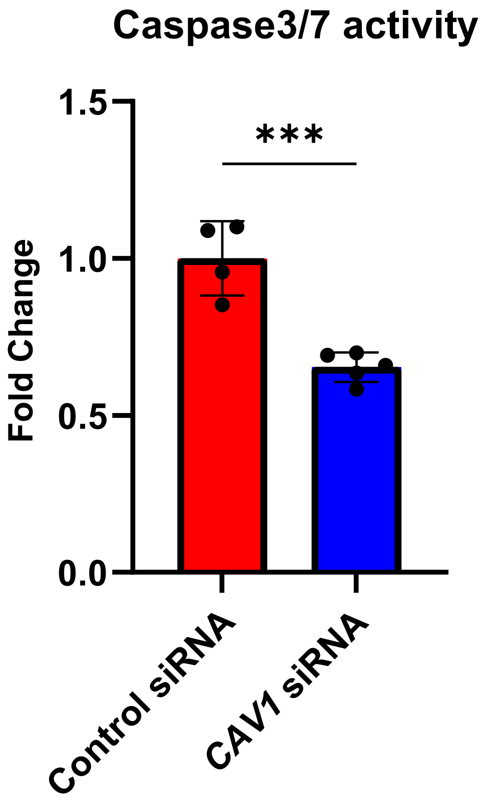

Supplementary Figure 6. CAV1-knockdown hPAECs are resistance to hypoxia-induced apoptosis.

After hPAECs were transfected with control siRNA or CAV1-specific siRNA, hypoxia stimulation with ischemic buffer was induced for 2 hr. Apoptosis of PAECs were evaluated with caspase 3/7 activity assays. ***P<0.001 compared with control siRNA. The data are shown as means ± sem.

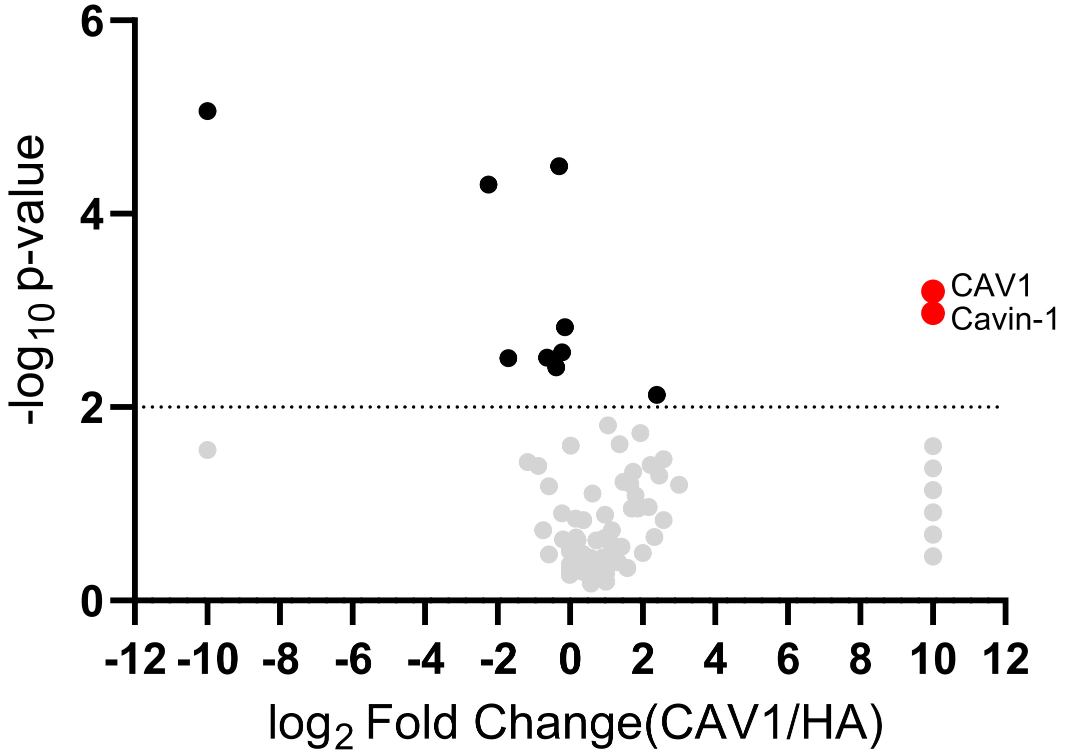

Supplementary Figure 7. Identified proteins by the screening with BioID that interact with CAV1 in hPAECs.

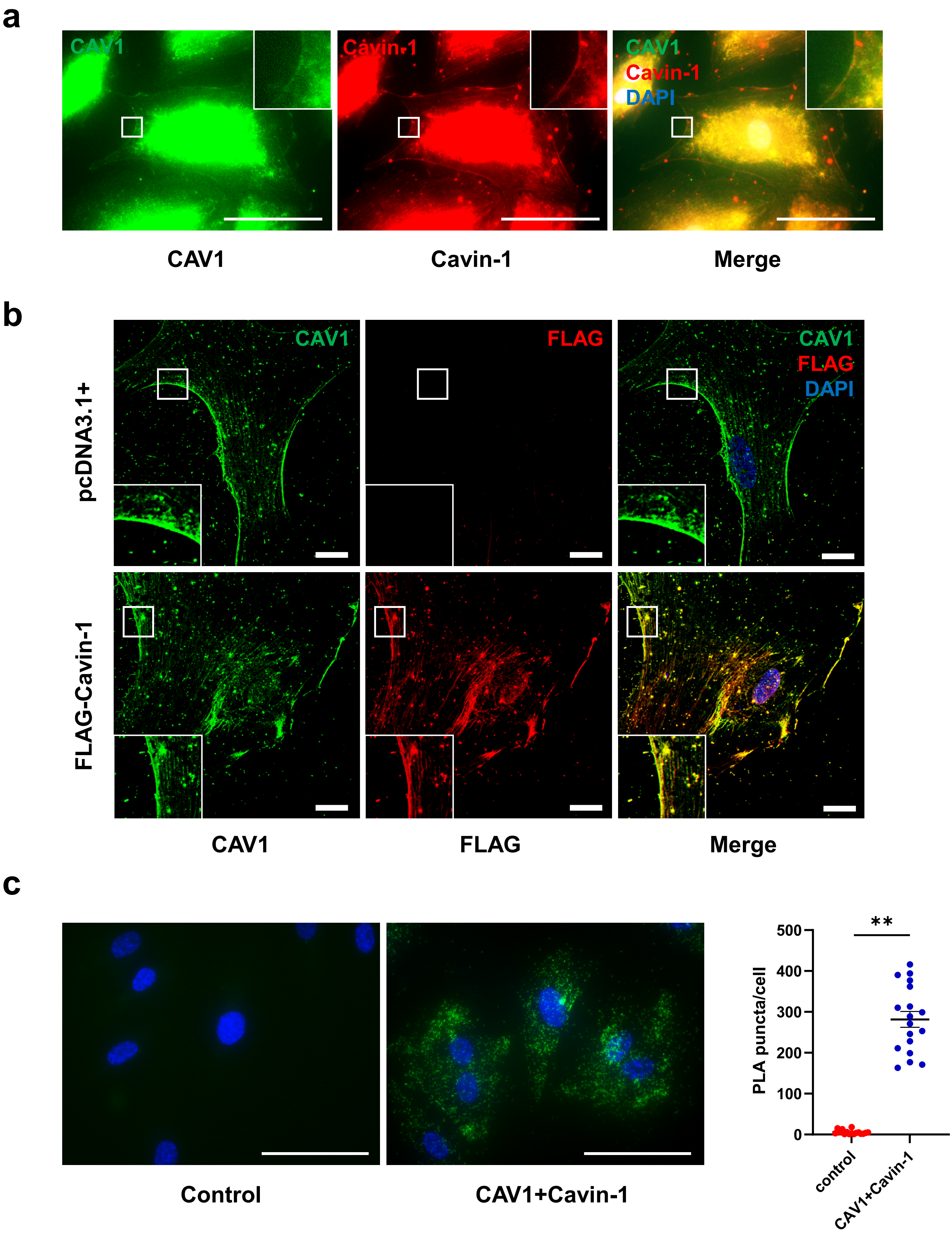

Supplementary Figure 8. Cavin-1 is associated with CAV1 in hPAECs.
(a) hPAECs was immunostained with anti-CAV1 and anti-Cavin-1 antibodies. Scale Bar, 50 μm. (b) hPAECs transfected with pcDNA3.1+ or pFLAG-CMV-4-hCavin-1 were immunostained with anti-CAV1 and anti-FLAG antibodies. Scale bar, 20 μm. (c) Representative fluorescent images of the PLA for the interaction between CAV1 and Cavin-1 in hPAECs. **P<0.01 compared with control. The data are shown as means ± sem. Scale bar, 50 μm.

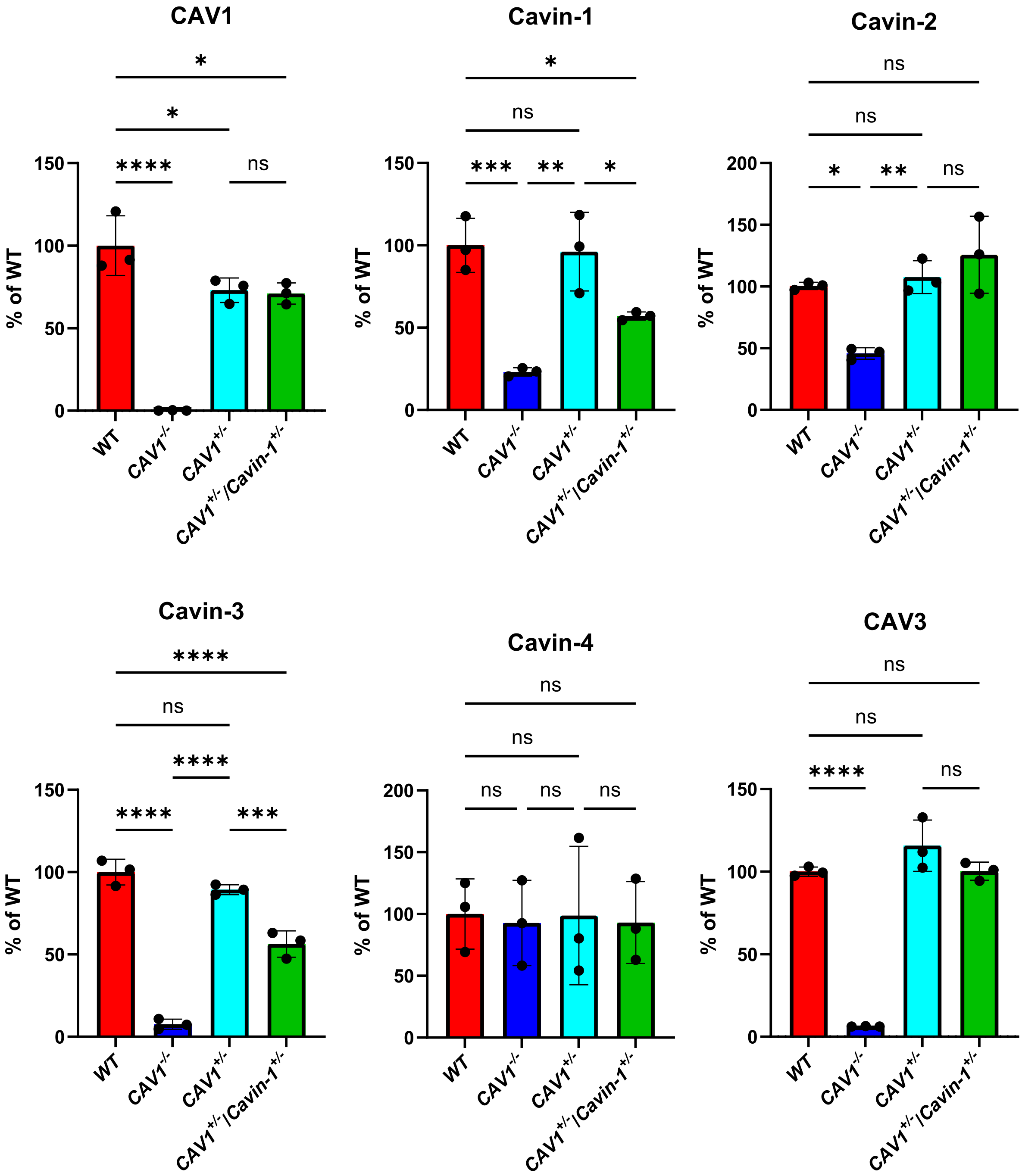

Supplementary Figure 9. Quantification of expression of caveolae-related proteins in the lung of WT, *CAV1*$^{-/-}$, *CAV1*$^{+/-}$, and *CAV1*$^{+/-}$/*Cavin-1*$^{+/-}$ mice.

Caveolae-related proteins in the lung of WT, *CAV1*$^{-/-}$, *CAV1*$^{+/-}$, and *CAV1*$^{+/-}$/*Cavin-1*$^{+/-}$ mice were assessed by Western blotting. *P<0.05, **P<0.01, ***P<0.001, and ****P<0.0001 between each group. The data are shown as means ± sem. ns, not significant.

Fig. 1b

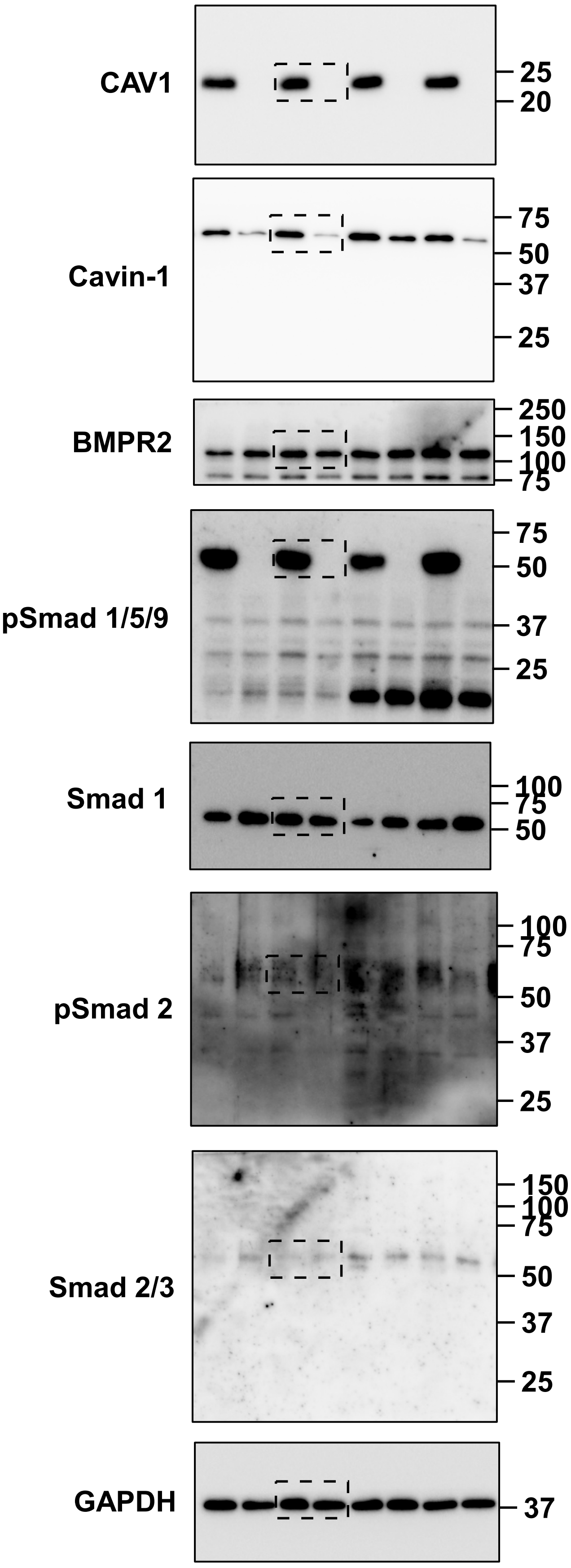

Fig. 1c

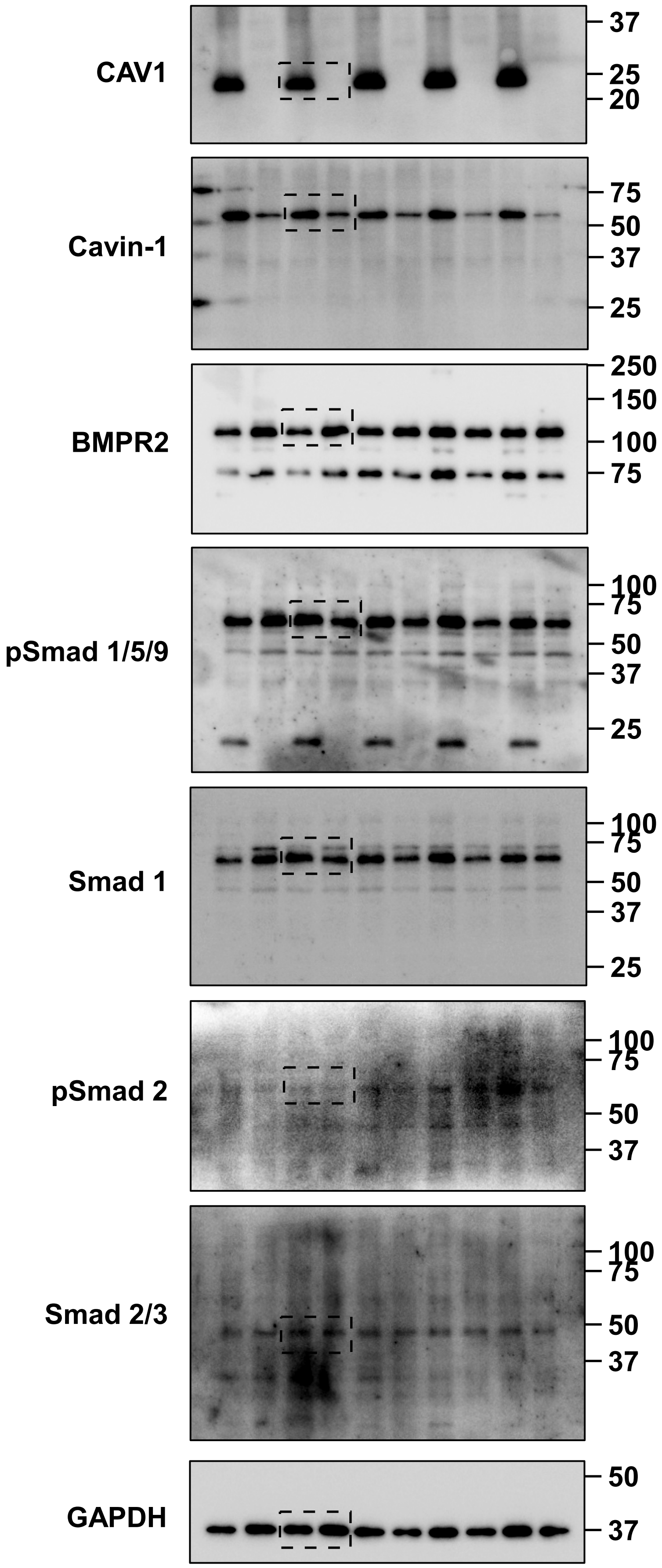

Supplementary Figure 10. Uncropped blots in main and supplementary figures. Uncropped Western blot images are shown that correspond to Fig. 1.

Fig. 2a

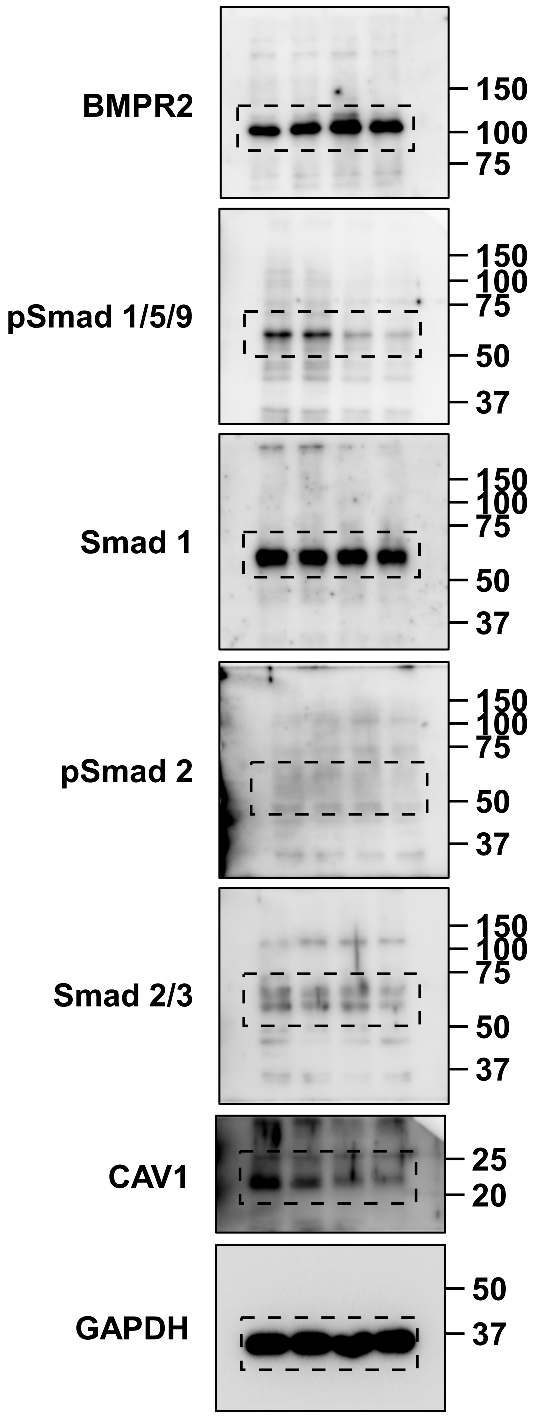

Fig. 2b

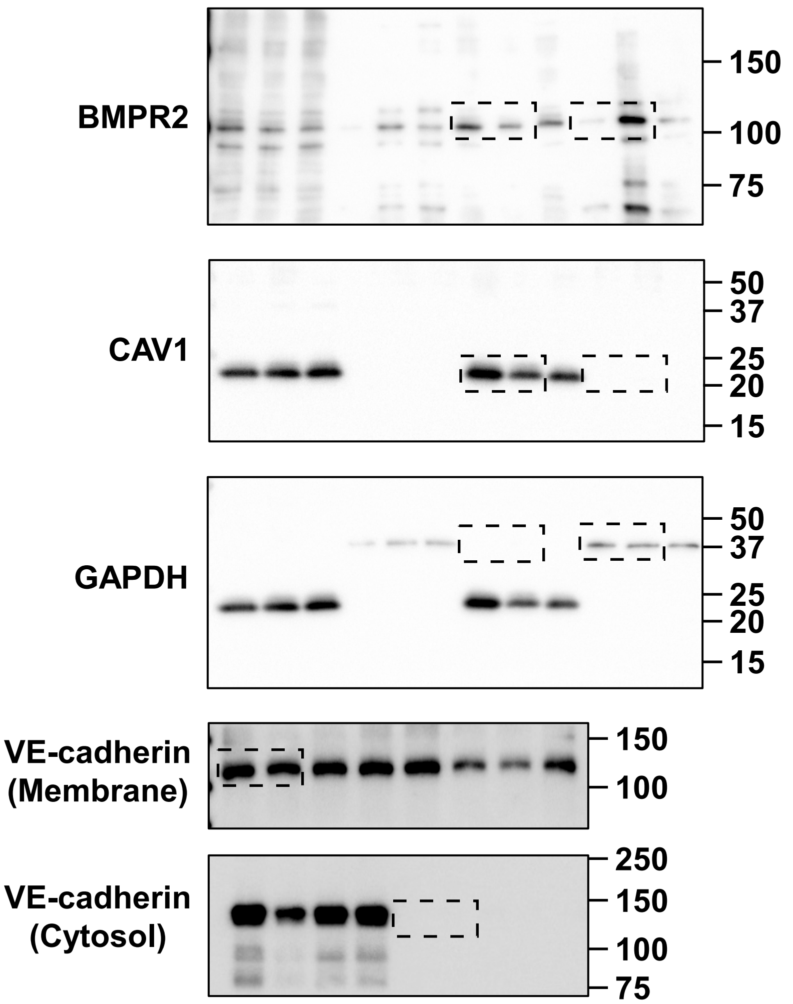

Supplementary Figure 10. Uncropped blots in main and supplementary figures (Continued). Uncropped Western blot images are shown that correspond to Fig. 2.

Fig. 3a

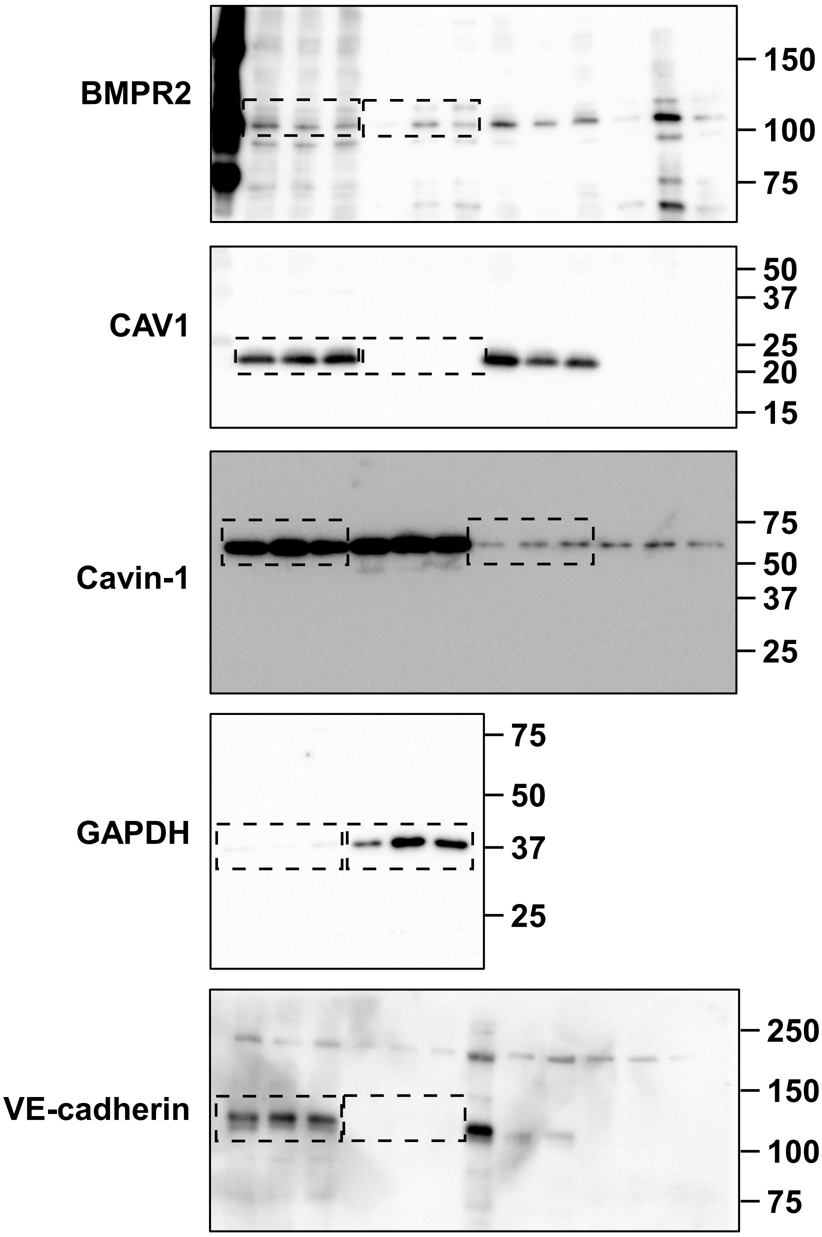

Supplementary Figure 10. Uncropped blots in main and supplementary figures (Continued). Uncropped Western blot images are shown that correspond to Fig. 3.

Fig. 4a

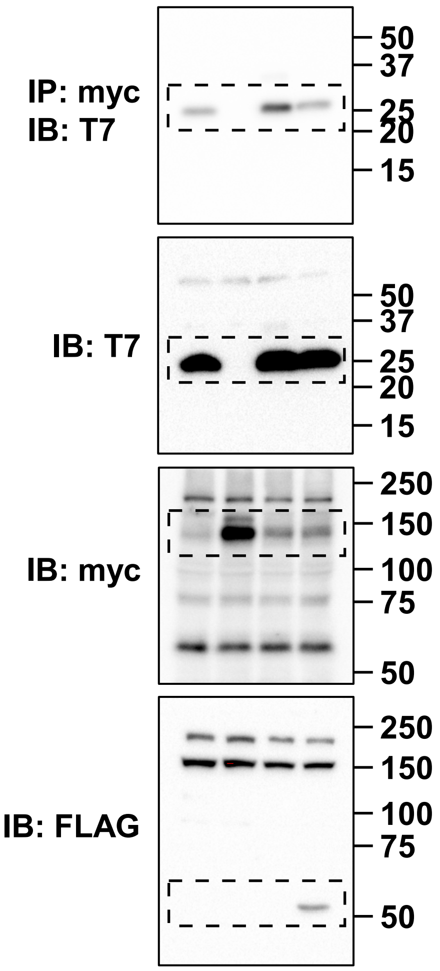

Fig. 4d

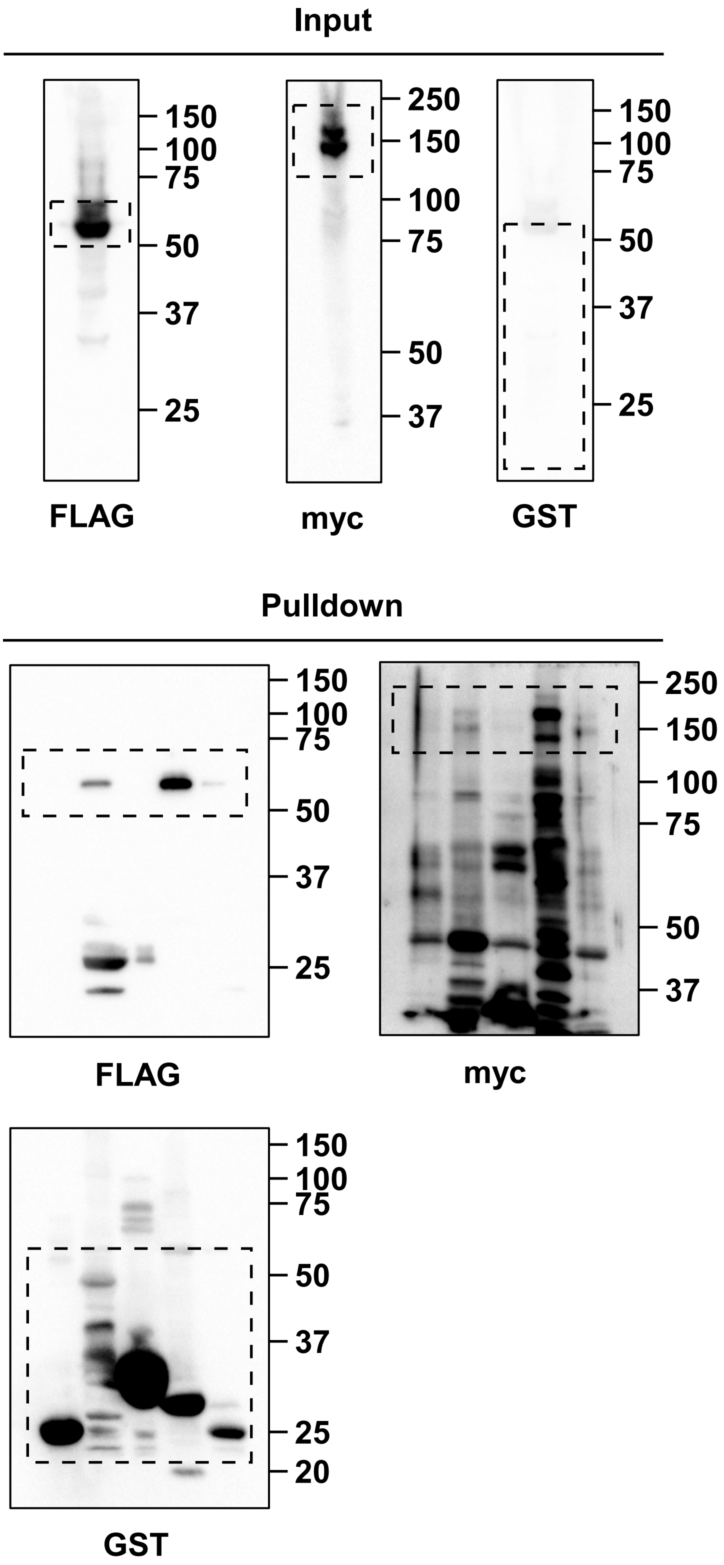

Supplementary Figure 10. Uncropped blots in main and supplementary figures (Continued). Uncropped Western blot images are shown that correspond to Fig. 4.

Fig. 4e

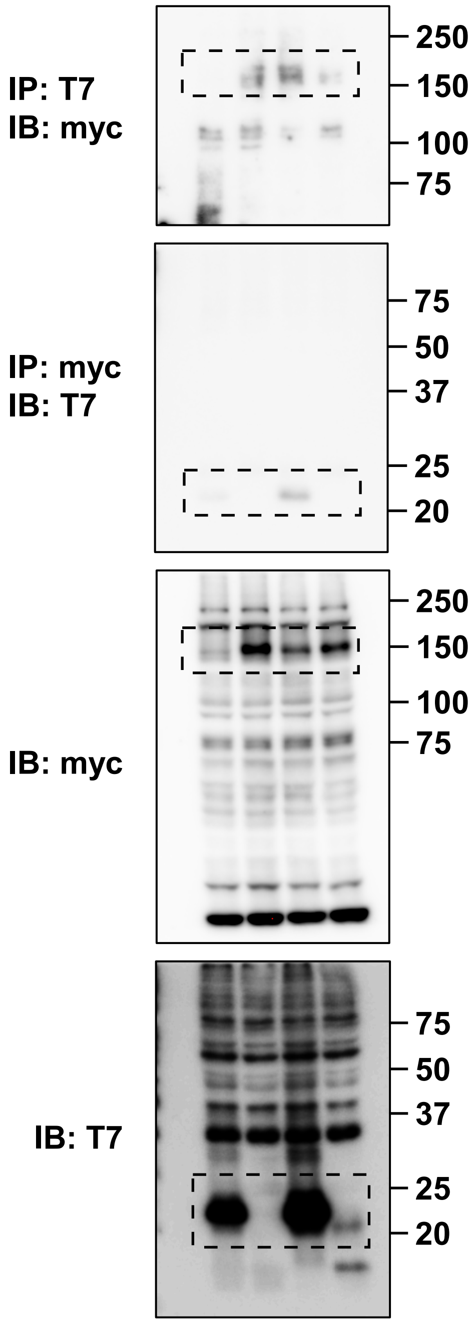

Fig. 4f

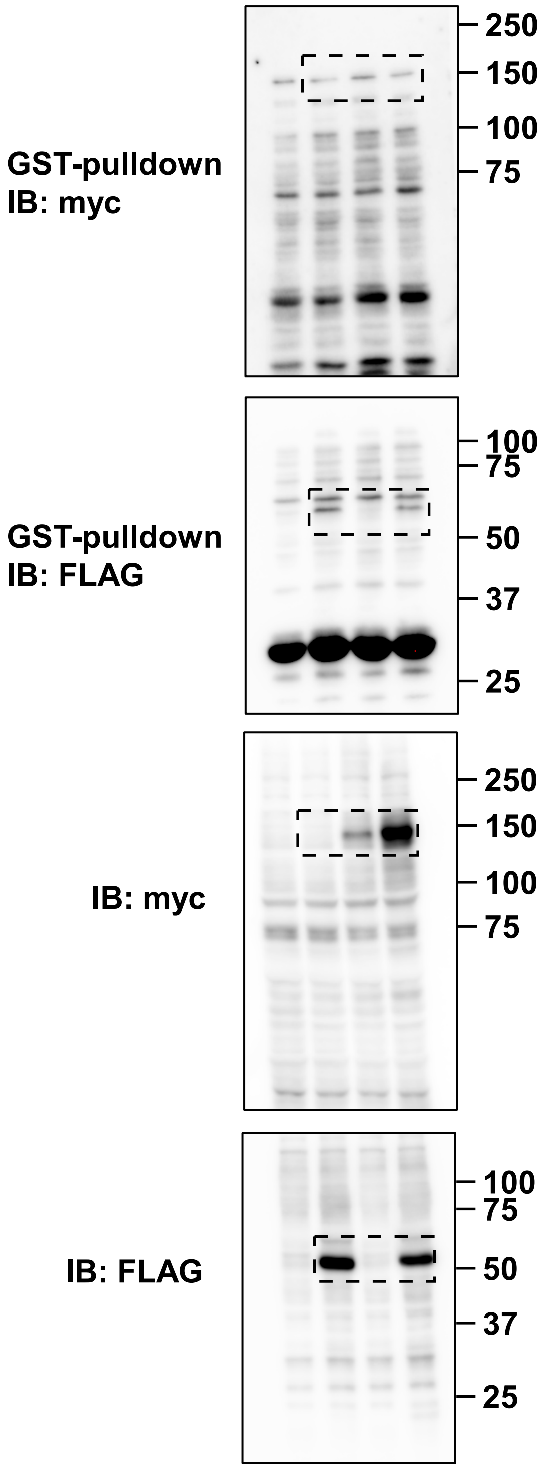

Fig. 4e

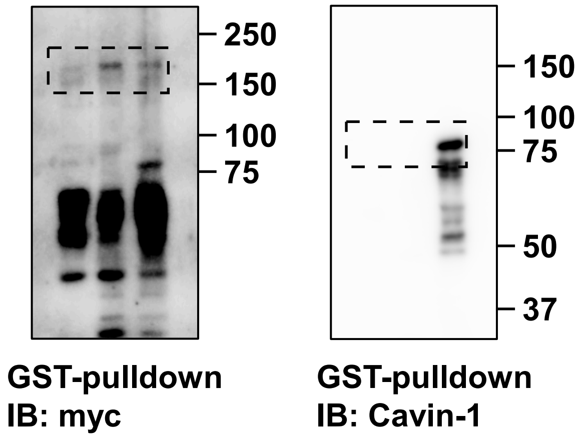

Supplementary Figure 10. Uncropped blots in main and supplementary figures (Continued). Uncropped Western blot images are shown that correspond to Fig. 4.

Fig. 5a

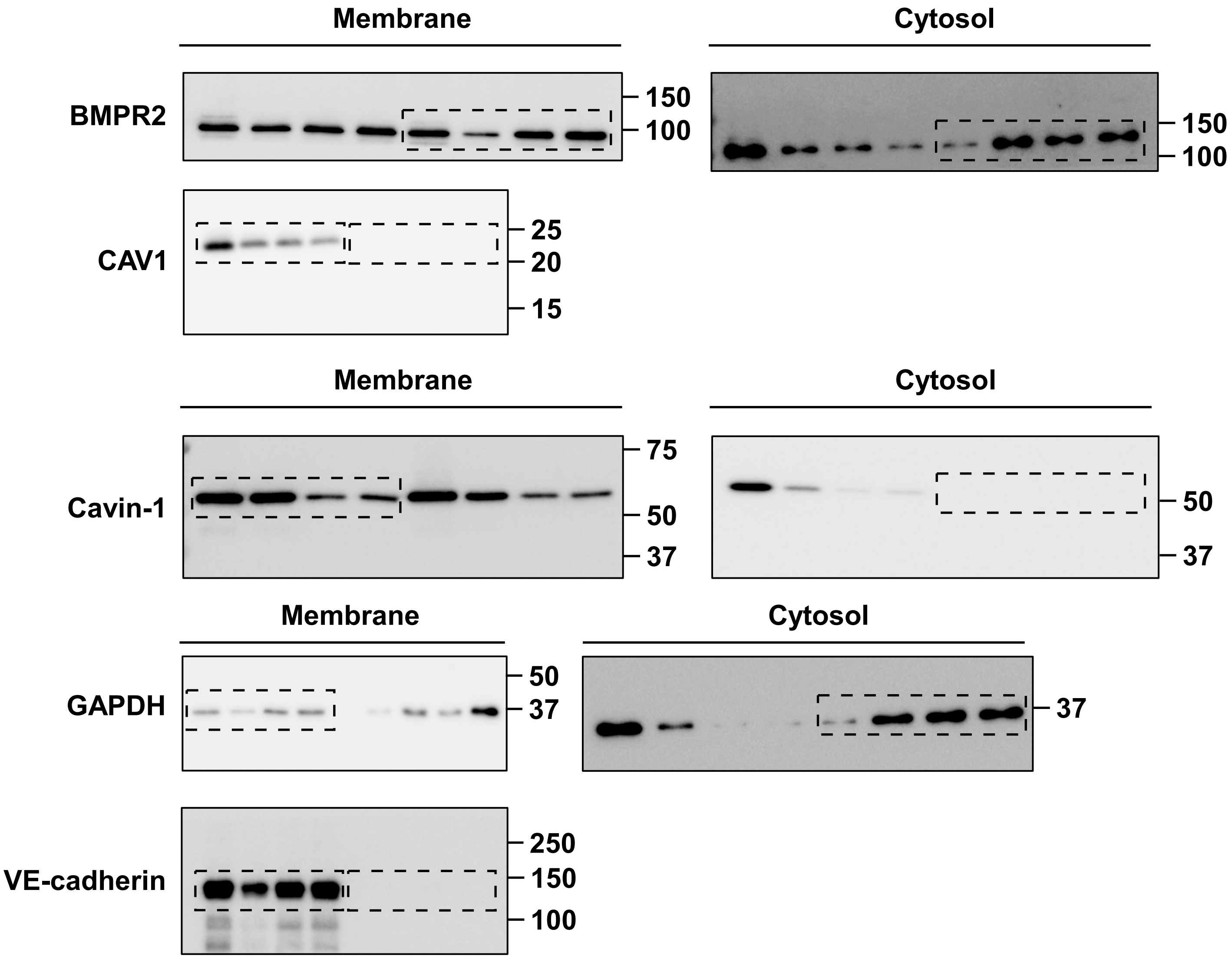

Fig. 5c

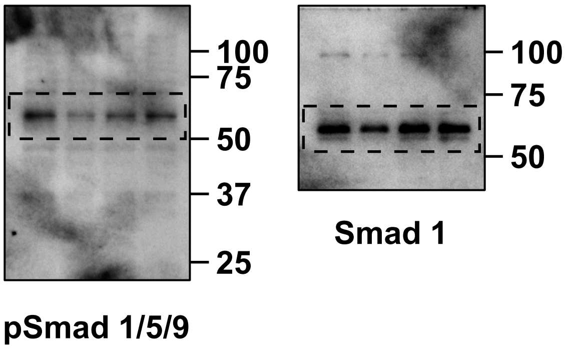

Supplementary Figure 10. Uncropped blots in main and supplementary figures (Continued). Uncropped Western blot images are shown that correspond to Fig. 5.

Fig. 6a

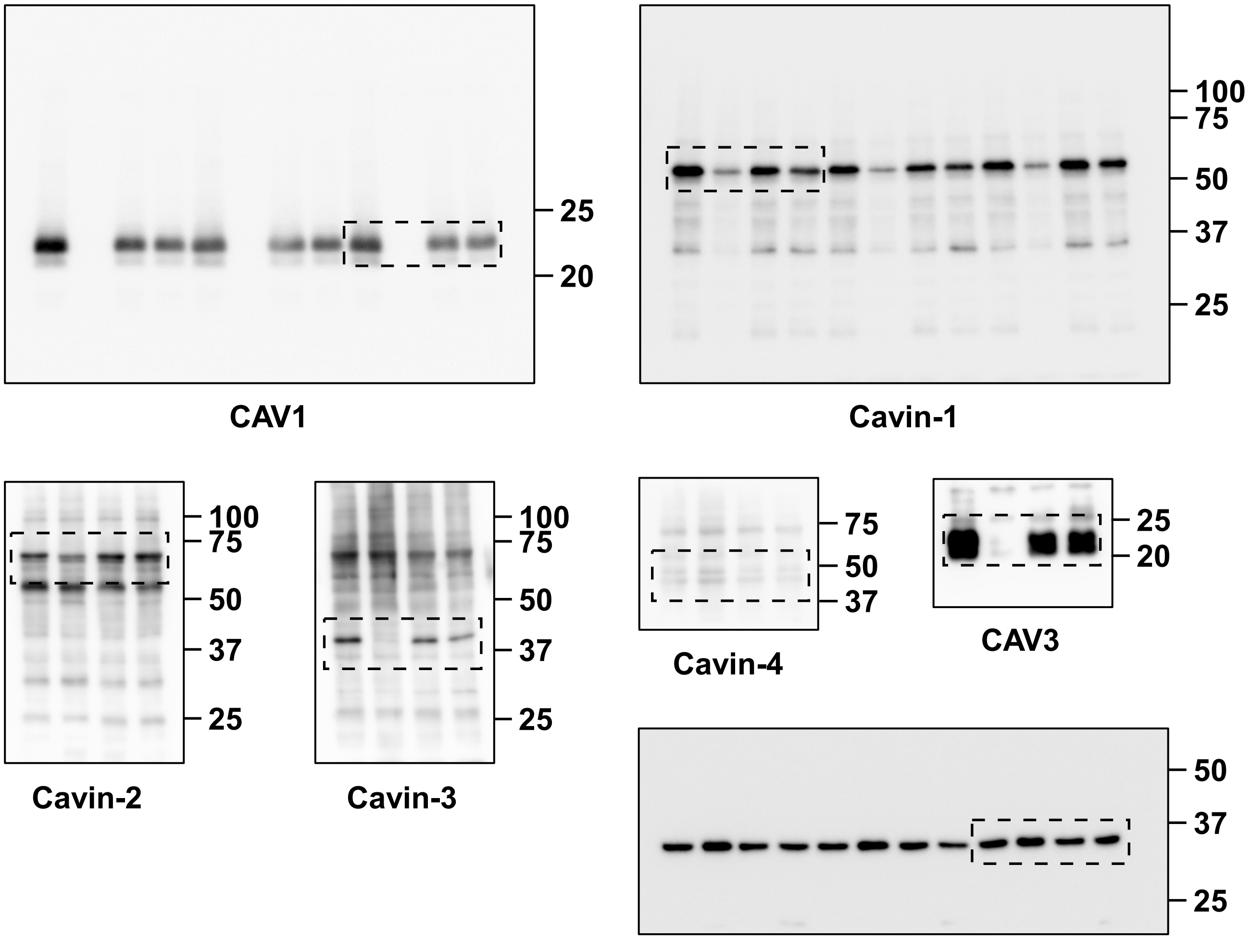

Fig. 6a

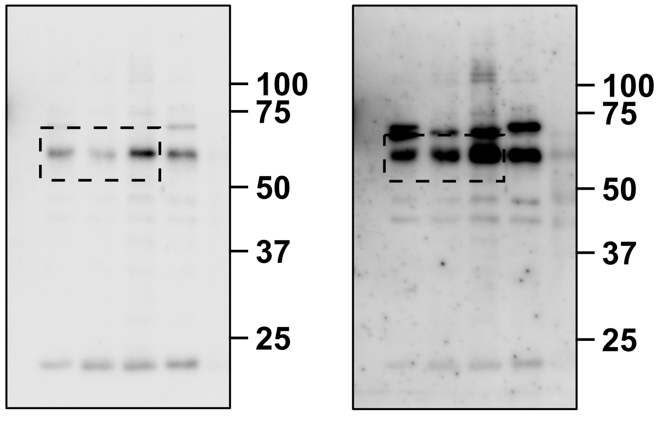

Supplementary Figure 10. Uncropped blots in main and supplementary figures (Continued). Uncropped Western blot images are shown that correspond to Fig. 6.

Fig. S1d

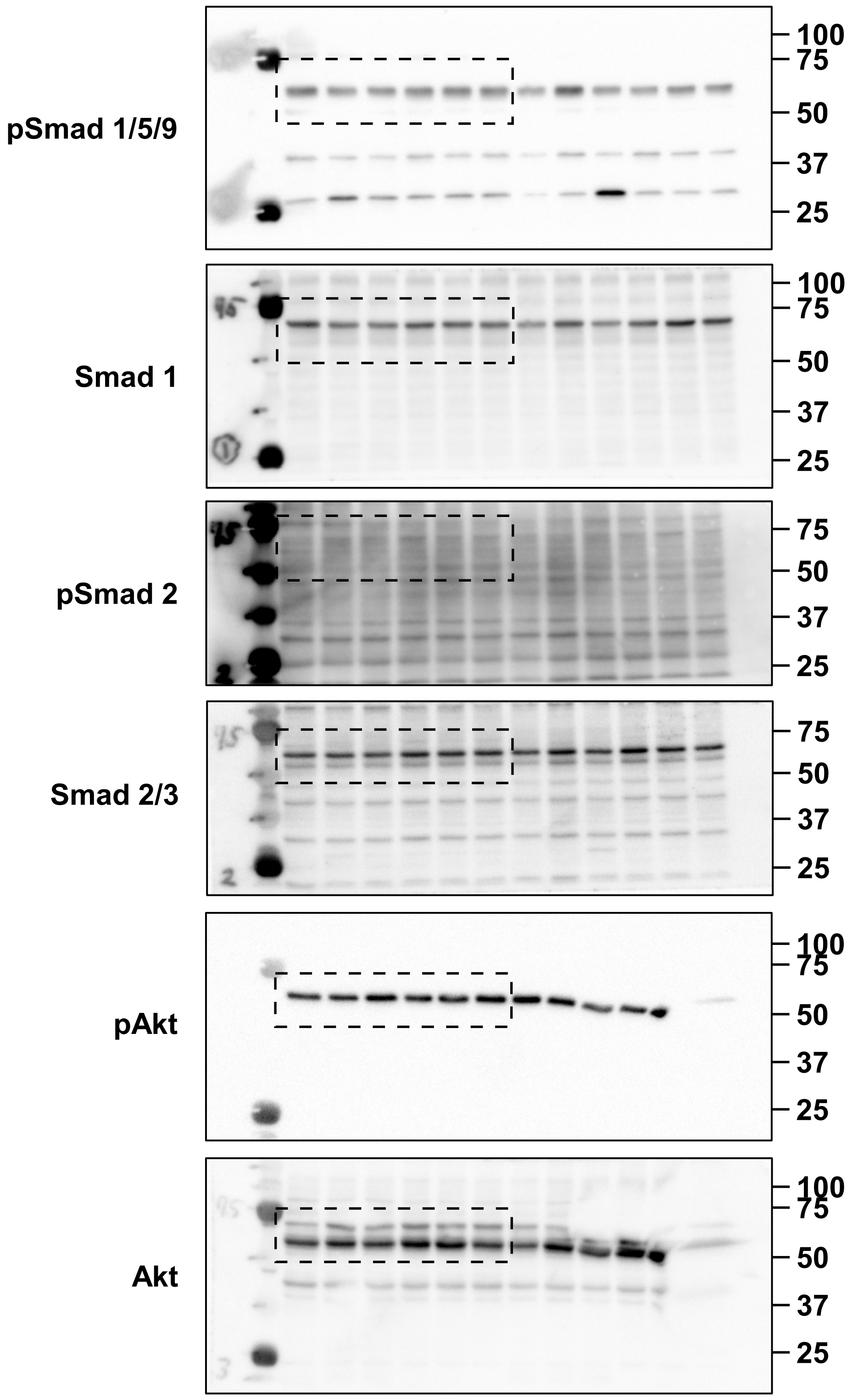

Supplementary Figure 10. Uncropped blots in main and supplementary figures (Continued). Uncropped Western blot images are shown that correspond to Fig. S1.

Fig. S4a

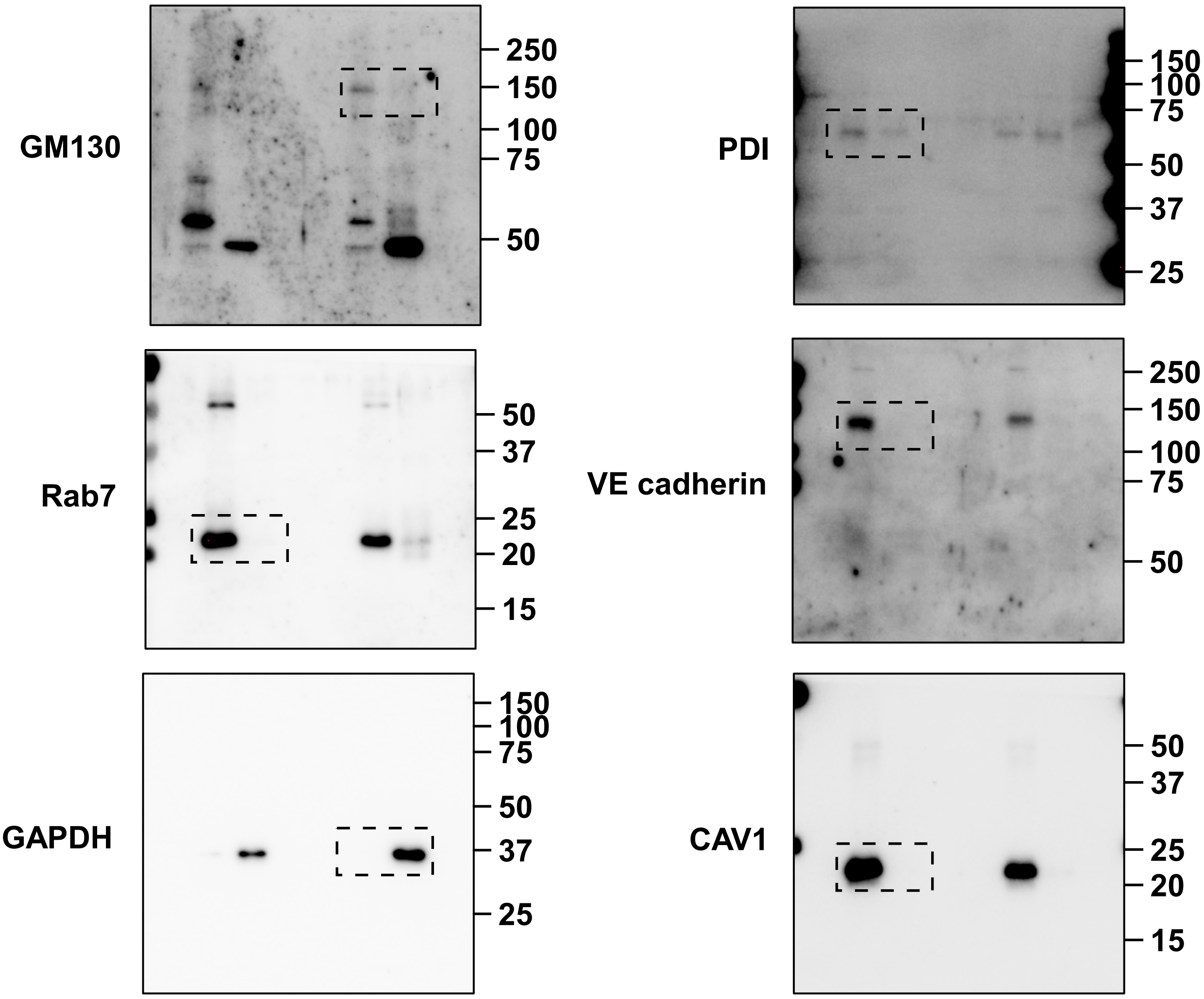

Supplementary Figure 10. Uncropped blots in main and supplementary figures (Continued). Uncropped Western blot images are shown that correspond to Fig. S4.
