# Supplementary material for: The Cavin-1/Caveolin-1 interaction attenuates BMP/Smad signaling in pulmonary hypertension by interfering with BMPR2/Caveolin-1 binding
Source: Commun Biol. 2024 Jan 5;7:40. doi: 10.1038/s42003-023-05693-2 (PMC10770141; doi:10.1038/s42003-023-05693-2)
Supplement: Supplementary file 5 — Reporting Summary [file 42003_2023_5693_MOESM5_ESM.pdf]

nature portfolio

Corresponding author(s): Naohiko Nakanishi

Last updated by author(s): Sep 18, 2023

Reporting Summary

Nature Portfolio wishes to improve the reproducibility of the work that we publish. This form provides structure for consistency and transparency in reporting. For further information on Nature Portfolio policies, see our Editorial Policies and the Editorial Policy Checklist.

Statistics

For all statistical analyses, confirm that the following items are present in the figure legend, table legend, main text, or Methods section.

n/a	Confirmed	
☐	☒	The exact sample size (*n*) for each experimental group/condition, given as a discrete number and unit of measurement
☐	☒	A statement on whether measurements were taken from distinct samples or whether the same sample was measured repeatedly
☐	☒	The statistical test(s) used AND whether they are one- or two-sided<br>*Only common tests should be described solely by name; describe more complex techniques in the Methods section.*
☐	☒	A description of all covariates tested
☐	☒	A description of any assumptions or corrections, such as tests of normality and adjustment for multiple comparisons
☐	☒	A full description of the statistical parameters including central tendency (e.g. means) or other basic estimates (e.g. regression coefficient) AND variation (e.g. standard deviation) or associated estimates of uncertainty (e.g. confidence intervals)
☐	☒	For null hypothesis testing, the test statistic (e.g. *F*, *t*, *r*) with confidence intervals, effect sizes, degrees of freedom and *P* value noted<br>*Give P values as exact values whenever suitable.*
☒	☐	For Bayesian analysis, information on the choice of priors and Markov chain Monte Carlo settings
☒	☐	For hierarchical and complex designs, identification of the appropriate level for tests and full reporting of outcomes
☒	☐	Estimates of effect sizes (e.g. Cohen's *d*, Pearson's *r*), indicating how they were calculated

Our web collection on statistics for biologists contains articles on many of the points above.

Software and code

Policy information about availability of computer code

Data collection	Fluorescent signals were detected using a Keyence BZ-X700 digital microscope (Osaka, Japan) or Zeiss LSM510 META Confocal Imaging System (Oberkochen, Germany). PLA signal was captured using a Keyence BZ-X700 digital microscope. For the transmission electron microscopy, microtome sections were examined under an H-7100 transmission electron microscope (HITACHI, Tokyo, Japan). luminescence in apoptosis assay was measured using a TECAN microplate reader (Zurich, Switzerland). To measure RV hemodynamics, open-chest RV catheterization using a 1.2-F pressure catheter (Transonic Scisense, Inc., London, ON, Canada) was performed. LC–MS/MS analyses were performed using a nano LC (UltiMate® 3000) (Dionex, Sunnyvale, CA, USA) coupled with a Q Exactive Plus Orbitrap mass spectrometer (Thermo Scientific, Waltham, MA, USA). Instrument operation and data acquisition were performed using Xcalibur Software (Thermo Scientific, Waltham, MA, USA).
Data analysis	The MS/MS raw data were processed using Mascot version 2.6.0 (Matrix Sciences, London, US) and were searched in the Swiss-Prot database with humans as the species, carbamidomethylation of cysteine as a static modification, oxidation of methionine as a dynamic modification, precursor mass tolerance of 1.0 Da, and a fragment mass tolerance of 0.8 Da. The dat files of all fractions obtained were processed with Scaffold version 5.0.1 (Proteome Software Inc.). All statistical analyses were performed using GraphPad Prism 8 (GraphPad Software, Inc., CA, USA).

For manuscripts utilizing custom algorithms or software that are central to the research but not yet described in published literature, software must be made available to editors and reviewers. We strongly encourage code deposition in a community repository (e.g. GitHub). See the Nature Portfolio guidelines for submitting code & software for further information.

Data

Policy information about availability of data

All manuscripts must include a data availability statement. This statement should provide the following information, where applicable:

- Accession codes, unique identifiers, or web links for publicly available datasets
- A description of any restrictions on data availability
- For clinical datasets or third party data, please ensure that the statement adheres to our policy

The authors declare that all data supporting the findings of this study are available within the article and its Supplementary Information Files and from the authors upon request.

Human research participants

Policy information about studies involving human research participants and Sex and Gender in Research.

Reporting on sex and gender	N/A
Population characteristics	N/A
Recruitment	N/A
Ethics oversight	N/A

Note that full information on the approval of the study protocol must also be provided in the manuscript.

Field-specific reporting

Please select the one below that is the best fit for your research. If you are not sure, read the appropriate sections before making your selection.

☒ Life sciences ☐ Behavioural & social sciences ☐ Ecological, evolutionary & environmental sciences

For a reference copy of the document with all sections, see nature.com/documents/nr-reporting-summary-flat.pdf

Life sciences study design

All studies must disclose on these points even when the disclosure is negative.

Sample size	The sample size for in vivo studies were determined based on our preliminary data. The sample size was selected to produce statistically relevant biological difference in the study. Sample sizes were determined in accordance with the literature and based on previous experience in our group.
Data exclusions	No data were excluded from the analyses.
Replication	The experiments were performed at least three times independently.
Randomization	Randomizations of the mice were performed based on the age and genotype. Littermates were used for each experiment.
Blinding	Measurement of pressure and histological analyses were performed in an semi-blinded manner, by assigning each mouse a number at the start of the experiment. The samples were analyzed only by mouse number.

Reporting for specific materials, systems and methods

We require information from authors about some types of materials, experimental systems and methods used in many studies. Here, indicate whether each material, system or method listed is relevant to your study. If you are not sure if a list item applies to your research, read the appropriate section before selecting a response.

Materials & experimental systems

n/a	Involved in the study
	☒ Antibodies
	☒ Eukaryotic cell lines
☒	☐ Palaeontology and archaeology
	☒ Animals and other organisms
☒	☐ Clinical data
☒	☐ Dual use research of concern

Methods

n/a	Involved in the study
☒	☐ ChIP-seq
☒	☐ Flow cytometry
☒	☐ MRI-based neuroimaging

Antibodies

Antibodies used

The rabbit polyclonal anti-CAV1 antibody, the mouse monoclonal anti-CAV1 antibody, the monoclonal anti-BMPR1a antibody and the mouse monoclonal anti-VE cadherin antibody were purchased from Santa Cruz Biotechnology (Dallas, TX, USA); the rabbit polyclonal antibody to Cavin-1 and αSMA, the horseradish peroxidase-conjugated monoclonal antibody to GAPDH and the mouse monoclonal antibody to myc tag were from Abcam PLC (Cambridge, UK); the rabbit polyclonal antibodies to Akt, phospho-Akt (Ser473), phospho-Smad1 (Ser463/465)/Smad5 (Ser463/465)/Smad9 (Ser465/467), Smad1, phospho-Smad2 (Ser465/467), Smad2/3 and the horseradish peroxidase-conjugated secondary antibodies (anti-mouse-HRP, and anti-rabbit-HRP) were purchased from Cell Signaling Technology (Danvers, MA, USA); rabbit polyclonal antibody to Cavin-2 and Cavin-3 (PRKCDBP) was purchased from ProteinTech Group, Inc. (Rosemont, IL, USA); the rabbit polyclonal anti-Cavin-4 antibody was generated in our group; the mouse monoclonal antibody to FLAG (clone M2) and Cy3-conjugated monoclonal antibody to αSMA were from Merck (Darmstadt, Germany); the mouse monoclonal antibodies to CAV3 and the rat monoclonal antibodies to CD31 (clone MEC13.3) were from BD Biosciences (Franklin Lakes, NJ, USA); the horseradish peroxidase-conjugated monoclonal antibody to GST was from Wako Pure Chemical Industries (Osaka, Japan); the mouse monoclonal antibody to T7 was from Novus Biologicals (Colorado, USA). The mouse monoclonal antibody to BMPR2 (3F6F8, MA5-15827) was obtained from Thermo Fisher Scientific Inc. (Waltham, MA, USA).

Validation

1. Rabbit polyclonal anti-CAV1 antibody (N-20) (#sc-894, Santa Cruz Biotechnology, USA): https://www.scbt.com/p/caveolin-1-antibody-n-20
2. Mouse monoclonal anti-CAV1 antibody (4H312) (#sc-70516, Santa Cruz Biotechnology, USA): https://www.scbt.com/ja/p/caveolin-1-antibody-4h312
3. Mouse monoclonal anti-BMPR1a antibody (C7) (#sc-518037, Santa Cruz Biotechnology, USA): https://www.scbt.com/ja/p/bmpr-ia-antibody-c-7
4. Mouse monoclonal anti-VE cadherin antibody (F-8) (#sc-9989, Santa Cruz Biotechnology, USA): https://www.scbt.com/ja/p/ve-cadherin-antibody-f-8
5. Rabbit polyclonal anti-PTRF antibody (#ab76919, Abcam PLC, UK): https://www.abcam.co.jp/products/primary-antibodies/ptrf-antibody-ab76919.html
6. Rabbit polyclonal anti-αSMA antibody (#ab124964, Abcam PLC, UK): https://www.abcam.co.jp/products/primary-antibodies/alpha-smooth-muscle-actin-antibody-epr5368-ab124964.html
7. The horseradish peroxidase-conjugated monoclonal anti-GAPDH antibody (#ab105428, Abcam PLC, UK): https://www.abcam.com/products/primary-antibodies/gapdh-antibody-6c5-loading-control-hrp-ab105428.html
8. Mouse monoclonal anti-myc tag antibody (#ab32, Abcam PLC, UK): https://www.abcam.com/products/primary-antibodies/myc-tag-antibody-9e10-ab32.html
9. Rabbit polyclonal anti-Akt antibody (#9272, Cell Signaling Technology, USA): https://www.cellsignal.com/products/primary-antibodies/akt-antibody/9272
10. Rabbit polyclonal anti-phospho-Akt antibody (Ser473) (#9271, Cell Signaling Technology, USA): https://www.cellsignal.com/products/primary-antibodies/phospho-akt-ser473-antibody/9271
11. Rabbit polyclonal anti-phospho-Smad1 (Ser463/465)/Smad5 (Ser463/465)/Smad9 (Ser465/467) antibody (#13820, Cell Signaling Technology, USA): https://www.cellsignal.jp/products/primary-antibodies/phospho-smad1-ser463-465-smad5-ser463-465-smad9-ser465-467-d5b10-rabbit-mab/13820
12. Rabbit polyclonal anti-Smad1 antibody (#9743, Cell Signaling Technology, USA): https://www.cellsignal.com/products/primary-antibodies/smad1-antibody/9743
13. Rabbit polyclonal anti-phospho-Smad2 (Ser465/467) antibody (#3108, Cell Signaling Technology, USA): https://www.cellsignal.jp/products/primary-antibodies/phospho-smad2-ser465-467-138d4-rabbit-mab/3108
14. Rabbit polyclonal anti-Smad2/3 antibody (#3102, Cell Signaling Technology, USA): https://www.cellsignal.com/products/primary-antibodies/smad2-3-antibody/3102
15. The horseradish peroxidase-conjugated secondary antibodies (anti-mouse-HRP) (#7076, Cell Signaling Technology, USA): https://www.cellsignal.jp/products/secondary-antibodies/anti-mouse-igg-hrp-linked-antibody/7076
16. The horseradish peroxidase-conjugated secondary antibodies (anti-rabbit-HRP) (#7074, Cell Signaling Technology, USA): https://www.cellsignal.com/products/secondary-antibodies/anti-rabbit-igg-hrp-linked-antibody/7074
17. Rabbit polyclonal anti-SDPR antibody (#12339-1-AP, ProteinTech Group, Inc., USA): https://www.ptglab.co.jp/Products/SDPR-Antibody-12339-1-AP.htm
18. Rabbit polyclonal anti-PRKCDBP antibody (#16250-1-AP, ProteinTech Group, Inc., USA): https://www.ptglab.co.jp/Products/PRKCDBP-Antibody-16250-1-AP.htm
19. Rabbit polyclonal anti-Cavin-4 antibody was generated in our group: https://www.tandfonline.com/doi/full/10.1128/MCB.02186-07
20. Mouse monoclonal anti-FLAG antibody (#F3165, clone M2, Merck, Germany): https://www.sigmaaldrich.com/JP/ja/product/sigma/f3165
21. Cy3-conjugated monoclonal anti-αSMA antibody (#C6198, Merck, Germany): https://www.sigmaaldrich.com/JP/ja/product/sigma/c6198
22. Mouse monoclonal anti-T7 antibody (#69522, Merck, Germany): https://www.sigmaaldrich.com/JP/ja/product/mm/69522
23. Mouse monoclonal anti-CAV3 antibody (#610421, BD Biosciences, USA): https://www.bdbiosciences.com/en-us/products/

reagents/microscopy-imaging-reagents/immunohistochemistry-reagents/purified-mouse-anti-caveolin-3.610421
24. Rat monoclonal anti-CD31 antibody (#550274, clone MEC13.3, BD Biosciences, USA): https://www.bdbiosciences.com/en-us/products/reagents/flow-cytometry-reagents/research-reagents/single-color-antibodies-ruo/purified-rat-anti-mouse-cd31.550274
25. The horseradish peroxidase-conjugated monoclonal anti-GST antibody (#NB600-388, Wako Pure Chemical Industries, Japan): https://labchem-wako.fujifilm.com/jp/product/detail/W01NVBNB600-388.html
26. Mouse monoclonal anti-BMPR2 antibody (#MA5-15827, 3F6F8, Thermo Fisher Scientific Inc., USA): https://www.thermofisher.com/antibody/product/BMPR2-Antibody-clone-3F6F8-Monoclonal/MA5-15827

Eukaryotic cell lines

Policy information about cell lines and Sex and Gender in Research

Cell line source(s)	Human PAECs (Catalog #: CC-2530) were purchased from Lonza (Walkersville, MD, USA). 293FT cells were kindful gift from Dr.Daisuke Kami.
Authentication	The cells were not authenticated.
Mycoplasma contamination	All cell lines were negative for Mycoplasma contamination.
Commonly misidentified lines (See ICLAC register)	None of the used cell lines are listed in ICLAC database.

Animals and other research organisms

Policy information about studies involving animals; ARRIVE guidelines recommended for reporting animal research, and Sex and Gender in Research

Laboratory animals	CAV1-knockout (CAV1-/-) mice (C57BL/6J background) and Cavin-1-knockout (Cavin-1-/-) mice (C57BL/6J background) were purchased from the Jackson Laboratory. Male mice aged 8–16 weeks were used in this study.
Wild animals	No wild animals were used in this study.
Reporting on sex	Both male and female mice were used in the experiments, allocating sex- and age-matched mice to each group. No apparent difference was observed in the analyses between male and female mice.
Field-collected samples	No field-collected samples were used in this study.
Ethics oversight	All animal care and experimentation procedures performed in this study were approved by the Institutional Animal Care and Use Committee of Kyoto Prefectural University of Medicine.

Note that full information on the approval of the study protocol must also be provided in the manuscript.
